# New tools provide a second look at HDV ribozyme structure, dynamics and cleavage

**DOI:** 10.1093/nar/gku992

**Published:** 2014-10-17

**Authors:** Gary J. Kapral, Swati Jain, Jonas Noeske, Jennifer A. Doudna, David C. Richardson, Jane S. Richardson

**Affiliations:** 1 Department of Biochemistry, Duke University, Durham, NC 27710, USA; 2 Program in Computational Biology and Bioinformatics, Duke University, Durham, NC 27710, USA; 3 Department of Molecular and Cell Biology, University of California Berkeley, Berkeley, CA 94720, USA

## Abstract

The hepatitis delta virus (HDV) ribozyme is a self-cleaving RNA enzyme essential for processing viral transcripts during rolling circle viral replication. The first crystal structure of the cleaved ribozyme was solved in 1998, followed by structures of uncleaved, mutant-inhibited and ion-complexed forms. Recently, methods have been developed that make the task of modeling RNA structure and dynamics significantly easier and more reliable. We have used ERRASER and PHENIX to rebuild and re-refine the cleaved and cis-acting C75U-inhibited structures of the HDV ribozyme. The results correct local conformations and identify alternates for RNA residues, many in functionally important regions, leading to improved R values and model validation statistics for both structures. We compare the rebuilt structures to a higher resolution, trans-acting deoxy-inhibited structure of the ribozyme, and conclude that although both inhibited structures are consistent with the currently accepted hammerhead-like mechanism of cleavage, they do not add direct structural evidence to the biochemical and modeling data. However, the rebuilt structures (PDBs: 4PR6, 4PRF) provide a more robust starting point for research on the dynamics and catalytic mechanism of the HDV ribozyme and demonstrate the power of new techniques to make significant improvements in RNA structures that impact biologically relevant conclusions.

## INTRODUCTION

Hepatitis D is a human disease that, in acute and chronic infections, can lead to increased chances of liver failure and liver cancer. It is caused by a small virus-like particle called hepatitis delta virus (HDV), which only infects patients who have a hepatitis B infection. HDV has a circular RNA genome of 1.7 kb that is replicated inside the host cells into genomic and antigenomic (complementary to the original genome) RNA. The replication is carried out by a rolling circle mechanism that produces a linear RNA strand containing multiple copies of the genome. These RNA strands are cleaved into single genome-length strands (which later ligate to form the circular genome), and this reaction also separates and processes the mRNA for the delta antigen, the only protein encoded by the HDV genome. This cleavage reaction is carried out by a stretch of 85 nucleotides (present both in genomic and antigenomic RNA) that form the self-cleaving RNA called the hepatitis delta virus ribozyme (1–3). The catalytic activity of the HDV ribozyme is essential for viral replication and viral particle assembly inside the host cells. HDV-like ribozymes have also been found in a variety of organisms and include the mammalian CPEB3 ribozyme found in the human CPEB3 gene known to play a role in human memory (4–7). Therefore, it is important to better understand the mechanism and structural basis of the self-cleaving activity of the HDV ribozyme.

The cleavage reaction generates a cyclic phosphate at the RNA product 3′ end and a hydroxyl group at its 5′ end, consistent with a nucleophilic attack on the scissile phosphate by the 2′ hydroxyl group of the previous residue (3). Either divalent or monovalent cations are required for the cleavage reaction to occur; however, the reaction rate in saturating Mg^2+^ is ∼3000 times higher than the reaction rate in 1 M Na^+^ (8,9). In addition, residue C75 (C76 in the antigenomic ribozyme) is essential for the cleavage reaction, and mutating C75 to any other base, especially uracil or guanine, inhibits the reaction (10–12). The 2.3Å resolution crystal structure of the genomic cleaved HDV ribozyme (13) revealed that C75 is located in the active site and near the 5′ hydroxyl, providing evidence that RNA residues can participate in general acid-base catalysis (a characteristic that was previously believed to be specific to proteins), a notion also supported by a study linking C75 to proton transfer (14). However, it is very difficult to distinguish unambiguously between a general-acid and general-base mechanism biochemically, and even more so structurally, and hence various studies have led to conflicting conclusions about the function of C75 in the cleavage reaction. Both the 2.3Å crystal structure of the cleaved ribozyme (13) and experiments using imidazole to restore cleavage in a C75U mutant ribozyme (15) suggested that C75 acts as a general base. Structures of the genomic cis-acting uncleaved ribozyme at resolutions ranging from 2.2 to 3.4Å, inhibited with a C75U mutation (16), also supported the general-base role for C75, with a proposed hinge rotation about the O3′-P bond to orient C75 and 2′ hydroxyl for the cleavage reaction. Other biochemical results supported a general-acid role for C75 (8) by demonstrating mutant ribozyme cleavage in a reaction buffer that substitutes for C75 and acts as a proton donor (17). Molecular dynamics simulations with the C75U-inhibited structure as a starting model also provided conflicting results. One study reported spontaneous rotation of the residue at position -1 (cleavage is between residues -1 and 1) to support the general-base role for C75 (18), and their efforts to simulate a general-acid role for C75 failed. Another study reported a similar spontaneous rotation, but supported the general-acid role for C75 (19).

**Figure 1.**
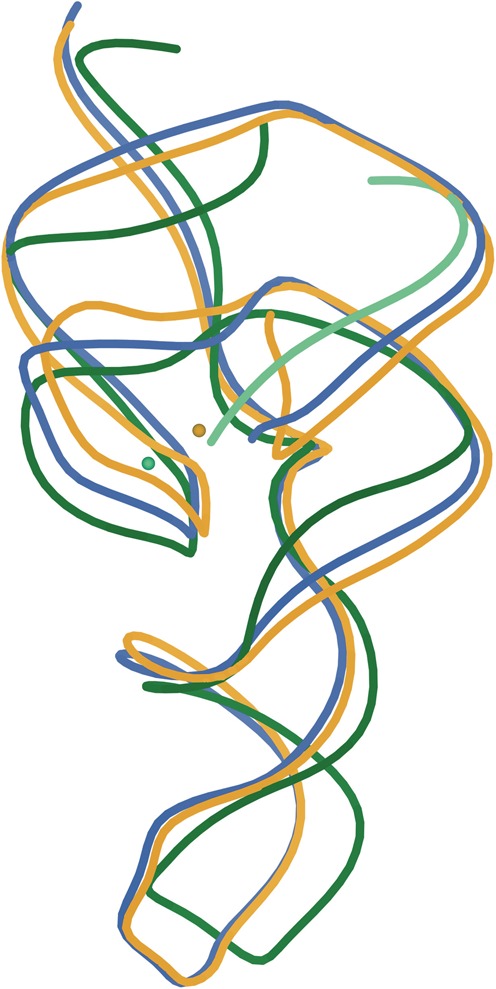
Superposition of the three crystal structures of the HDV ribozyme: rebuilt cis-acting C75U-inhibited (PDB ID: 4PRF in gold), rebuilt cleaved (PDB ID: 4PR6 in blue) and trans-acting deoxy-inhibited (PDB ID: 3NKB, ribozyme in green, substrate strand in light green). Active-site metal ions for the two inhibited structures are also shown in corresponding colors. The region surrounding the metal ions forms the active site.

**Figure 2.**
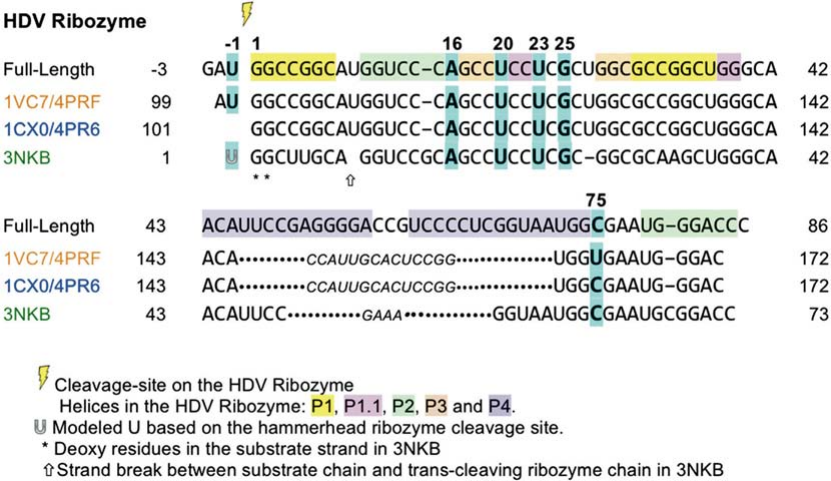
Sequences of the three HDV ribozyme crystal structures, aligned with the full-length ribozyme. The colors of the PDB IDs correspond to the colors of the splines in Figure 1. Residues discussed individually in the paper are highlighted in cyan and are in **bold**. Part of the P4 stem-loop was replaced by residues shown in *smaller italics* (U1A hairpin sequence motif in 1CX0/4PR6 and 1VC7/4PRF, and GAAA loop in 3NKB) to aid crystallization. These residues (146–159 in 1CX0/4PR6 and 1VC7/4PRF, 50–53 in 3NKB) do not have a corresponding sequence number in the full-length HDV ribozyme.

The most compelling evidence for a general-acid role for C75 was provided by mutations and single-atom substitutions on the catalytic pyrimidine and the leaving group, which associated the N3 atom of C75 with the leaving group (20). Additional support came from a later 1.9Å resolution crystal structure of the trans-acting HDV ribozyme (21), with the cleavage reaction inhibited by introducing deoxyribonucleotides at the scissile and neighboring position on the substrate strand. No significant electron density was observed for the single nucleotide upstream of the cleavage site. Therefore, to propose a model for the catalytic mechanism, the hammerhead ribozyme cleavage site (22) was superimposed on that of the HDV ribozyme, implying a similar reaction mechanism. This proposal is also consistent with a recent computational study that analyzes metal ion contributions to the C75 general-acid mechanism (23). Further biochemical studies comparing the pH-rate profiles of the wild-type and mutant ribozyme (24) and employing phosphorothioates at the scissile phosphate (25) also supported the general-acid role for C75. The current consensus in the field is that C75 acts as a general acid (26), with the hydrated metal ion acting as the general base.

One aspect which complicates resolving the catalytic mechanism of the HDV ribozyme is the substantial differences between constructs for the cleaved, the C75U-inhibited and the deoxy-inhibited crystal structures (compared in Figures 1 and 2), making clean interpretations difficult. Because the P4 stem-loop details are non-essential, crystallization was promoted by replacing its end (bottom of Figure 1) with known interaction motifs: the target sequence of the RNA-binding domain of spliceosomal protein U1A (U1A-RBD) in the early structures, and a GNRA tetraloop in the deoxy-inhibited structure. The sequence gap in the trans-acting deoxy-inhibited form shifts the P1 and P2 stems somewhat (green spline, Figure 1), and it also contains an additional base pair in the P2 stem. Of necessity, significant changes must be made to the molecule in order to prevent catalysis, especially on the crystallographic time scale. In addition, the early structures have areas of implausible RNA conformations around the active site. Now we are able to relieve that last problem.

Determining crystal structures of RNA, especially obtaining full-detail accuracy for the backbone with its many parameters per nucleotide (27,28), is in general a challenging task. Using recent technical developments not available for the early structures, both manual and automated tools have been developed that make the process much easier and more accurate. A set of 54 valid RNA backbone conformers, a reliable way to assign ribose pucker from clear observables in the electron density, and then use of pucker-specific target values for refinement (29–31) first enabled RNA Rotator (32) and RCrane (33) tools for manual fitting and the RNABC tool for automated backbone correction (34). Most importantly, there is now an automated, exhaustive local sampling system, called ERRASER (35–37) that extends and combines capabilities in the Rosetta (38), MolProbity (39) and PHENIX (29) software packages to correct nearly all local geometrical, conformational and steric errors in RNA structures. In the present study, we used the above new techniques to rebuild and re-refine the cleaved (PDB ID: 1CX0) (13) and the cis-acting C75U-inhibited (PDB ID: 1VC7) (16) structures of the HDV ribozyme. That process improved structural models for the HDV ribozyme active site and identified alternate conformations that fit the electron density better for some residues. The rebuilt structures have been deposited with the Protein Data Bank IDs of 4PR6 (cleaved) and 4PRF (C75U-inhibited). We also compare these rebuilt structures with a higher resolution structure of the trans-acting deoxy-inhibited ribozyme (PDB ID: 3NKB) (21) and highlight the similarities and the differences between them. Overall, our changes and corrections alter some of the biological interpretations about the catalytic mechanism that were based on the original structures, and our comparisons identify the lack of direct structural evidence for the position of upstream nucleotides preceding the cleavage reaction.

## MATERIALS AND METHODS

### Structure validation

To assess model-to-data match, we used *R*, *R*_free_ and difference electron density. To assess model quality, we used MolProbity (39,40) (version 4.1), a structure-validation web service that evaluates many features of RNA and protein models, or the equivalent functionality within PHENIX (29,41). MolProbity adds hydrogen atoms to the model, analyzes all-atom contacts and reports impossible steric clashes (atomic overlaps of >0.4Å) both individually and as ‘clashscore’. Any bond-lengths and bond-angles that deviate by >4*σ* from the expected values are flagged as outliers. Validation of protein residues includes evaluation for Ramachandran outliers, deviations in Cβ position and side-chain rotamer outliers.

MolProbity's RNA-specific evaluations include analysis of RNA ribose sugar pucker and RNA backbone conformers. The ribose ring in RNA almost always has one of two puckers, C3′-endo or C2′-endo, with the mean value of the δ dihedral angle (C5′-C4′-C3′-O3′) being 84 and 147**°**, respectively. To validate the modeled pucker for the ribose ring of a given residue, the value of the δ dihedral angle is compared to the length of the perpendicular dropped from the 3′ phosphate to the extended line of the glycosidic bond vector (31). If the δ dihedral and the length of the perpendicular indicate different puckers, then the modeled pucker is almost certainly incorrect and is flagged as an outlier. In PHENIX, this diagnosis is used to set appropriate target values for the ribose ring and backbone dihedral angles, improving refinement behavior.

The RNA sugar-phosphate backbone, with its many degrees of freedom, has been shown to be rotameric (27), and is most likely to adopt one of the known 54 backbone conformers (30). These conformers represent the favorable conformations of the sugar-to-sugar unit of RNA backbone called a *suite*. Each backbone conformer has a two-character name; for example, **1a** represents the conformation of the RNA backbone in a standard A-form helix. For RNA backbone validation in MolProbity and PHENIX, automated backbone conformer assignment is done by Suitename, and if a suite does not belong to any of the 54 backbone conformers, it is flagged as an outlier, denoted as **!!**. For details on validation methods, refer to the Supplementary Information section S2.

### Structure rebuilding and correction

We used several tools available to rebuild and correct the RNA chain. The most powerful tool is Enumerative Real-space Refinement ASisted by Electron density under Rosetta, or ERRASER (35,36). Given a PDB file, ERRASER keeps only the RNA residues and rebuilds any with problems in a three-stage procedure. To begin with, ERRASER minimizes all torsion angles and all backbone bond-lengths and bond-angles in the model using the Rosetta high-resolution energy function (38), which includes an electron density correlation score to ensure that the new model is consistent with the experimental data. Next, PHENIX's (29) MolProbity-style RNA validation tools are used to identify geometry, pucker and unrecognized backbone conformations in the minimized model (see previous section on structure validation). These residues, as well as residues with large rms deviation (>2Å) between their original position and the minimized position, are then rebuilt one at a time through single-nucleotide stepwise assembly (SWA), an *ab-initio* method of building each residue by enumerating many conformations covering all build-up paths (42). SWA is alternated with Rosetta ‘relax’ refinement (38), usually for three cycles. After the rebuilding process, the new model is minimized again. ERRASER can also be used to rebuild a single residue at a time, in which case it returns the top 10 possible distinct conformations. For the purposes of this paper, we used both the general and single-residue rebuilding capabilities of ERRASER available in Rosetta3.5 (38).

For manual fitting and correction of the RNA structure, we used RCrane (33), a plugin available in Coot version 0.6 (43), and the RNA Rotator tool available in the visualization software KiNG (32). RCrane uses RNA backbone pseudo dihedrals (to classify the relative position of the C1′ atom and the phosphate group) as input and builds the rest of the backbone atoms in the electron density, keeping the inputs fixed. RNA Rotator allows the user to choose a backbone suite to manually edit all seven backbone and two χ dihedral angles. It also provides a list of the 54 backbone conformers for the user to choose from and interactive feedback on many validation scores; for instance, all-atom contact analysis and quality of the backbone conformer is provided in real-time.

For protein corrections, we used the side-chain rotator tool in KiNG, which allows the user to try a specific rotamer from an updated version of the penultimate amino acid rotamer library (44) and then adjust the side-chain χ dihedral angles, with interactive feedback for both all-atom contacts and rotamer quality. We also used the backrub tool (45) available in KiNG to manually adjust the protein backbone in certain cases where moving the side chain alone was not sufficient to fix the error. Similar to RNA Rotator, both these tools carry out all-atom contact analysis and other validation in real-time for interactive feedback.

Note that the initial process of rebuilding was done from the crystallographic data and generic knowledge of RNA and protein structure. Structure comparisons and interpretation in terms of the specific functional issues for this ribozyme were then done jointly with the Doudna and Cate groups, followed by the necessary further investigations.

### Structure refinement

The structures were refined against the diffraction data, both before and after each stage of the rebuilding process, with the PHENIX crystallography software, version 1.8.4-1496 (29,41). Coordinates, individual B-factors and occupancies were refined, the weight of geometry versus diffraction data terms was optimized and target parameter values were ribose-pucker specific.

### Residue numbers

Figure 2 shows the sequences in the three crystal structures (cis-acting C75U-inhibited 1VC7/4PRF, cleaved 1CX0/4PR6 and trans-acting deoxy-inhibited 3NKB) aligned with the sequence of the full-length HDV ribozyme. In this paper, all RNA residues are numbered according to the full-length ribozyme, unless otherwise specified. Refer to the figure for the corresponding PDB coordinate-file residue numbers.

## RESULTS

The correction process and its conclusions are described from the more minor and straightforward to the more extensive and unusual. They are then discussed in terms of their functional relevance.

### Rebuild of the cleaved 1CX0 structure

The 1CX0 crystal structure of the HDV ribozyme self-cleaved product (13) was one of the earliest ribozyme structures ever solved, at 2.3Å resolution, with *R* = 24.6% and *R*_free_ = 27.9% as calculated by PHENIX. Notably, it exploited the ability of U1A-RBD protein to form crystal contacts, thereby facilitating crystal growth, by removing part of the P4 stem-loop of the genomic HDV ribozyme and replacing it with a U1A hairpin. Over 15 years later, MolProbity statistics can now diagnose many potential improvements in local model detail. For the RNA chain, there are 8/72 ribose pucker outliers, 16/72 unrecognized backbone conformations (denoted by **!!**) and 28 bond angles with deviations >4*σ*. For the overall structure, the clashscore is 13.8, which is better than most at that resolution (83rd percentile) but could be improved along with other corrections.

By using a combination of PHENIX refinement, ERRASER corrections and hand rebuilding in Coot and in KiNG, we made multiple changes to the model that improved its agreement with the experimental data and its MolProbity statistics. All geometry and ribose pucker outliers identified by MolProbity were corrected as a result of PHENIX refinement and rebuilding using ERRASER. The pucker corrections were particularly impressive; hand refits and the use of RNABC (26) were initially successful in correcting only one of these ribose puckers, PDB residue 152, refitting the C3′-endo pucker as C2′-endo, while the other seven ribose puckers were each corrected to C2′-endo pucker only by using ERRASER. Correcting these ribose pucker outliers resulted in four suites (sugar-to-sugar unit of the RNA backbone used for validation) changing to recognized backbone conformers. In total, 12 suites were changed from the original model, some moved from **!!** to a recognized backbone conformer, and some moved from one conformer to another. Overall, the number of unrecognized backbone conformations reduced from 16 to 9 (see Supplementary Table S1).

#### U23 alternate conformation

**Figure 3.**
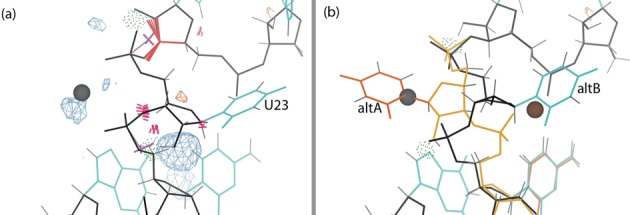
U23 and Mg^2+^ of the cleaved structure with Fo-Fc difference density at 3.5*σ* contour level. Here and in later figures, the blue and orange mesh correspond to positive and negative difference density respectively, hydrogen bonds are shown as green dotted pillows, steric clashes as hotpink spikes, ribose pucker outliers as magenta crosses, and bond-length or bond-angle outliers as springs or fans (blue if too small, red if too large). (**a**) The base and backbone of U23 in the original structure (1CX0), with the modeling errors highlighted. (**b**) Alternate conformations of U23 in the rebuilt structure (4PR6), with the backbone (gold), base (orange) and metal ion (brown) for the new conformation. All the modeling errors are corrected, and the difference density peaks have disappeared.

The electron density around residue U23 (Figure 3(a)) suggests a possible alternate conformation with the base facing the opposite direction to that of the original model. Indeed, cleavage-inhibited crystal structures show positions more like this second alternate. Given this evidence, we fit a second alternate, based loosely on PDB residues U23-C24 in 1SJ3 (16) and in 3NKB. The alternate was created in Coot and rebuilt in ERRASER before PHENIX refinement. After refinement, it was rebuilt in ERRASER a second time, using the single-residue rebuild option. The end result of this process has the density previously occupied by the Mg^2+^ in 1CX0 now occupied by the alternate conformation base (Figure 3(b)), with the N1 in nearly the same place as the original magnesium. A closer look at the new density showed that the alternate (alt A) should also have Mg^2+^ bound to the phosphate oxygen atoms of C24, approximately equivalently to Mg^2+^ bound to the original model's (alt B) conformation. Refinement shows this pair of alternates to fit the data well, lowering both *R* and *R*_free_. Occupancy refinement assigns alternate A and alternate B 0.64 and 0.36 occupancy, respectively. Neither alternate is a recognized backbone conformer, but they have no clashes or other outliers to show them invalid rather than just rare.

#### G25:U20 reverse wobble base pair

**Figure 4.**
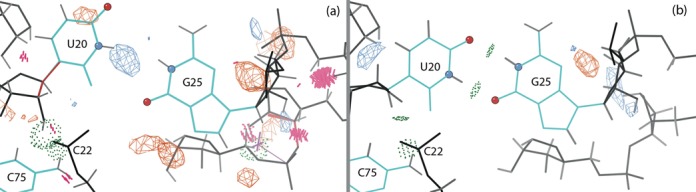
G25 and U20 in the cleaved ribozyme structure with Fo-Fc difference density at 3.5*σ* contour level. The hydrogen-bonded heavy atoms of U20 and G25 are highlighted as atom-colored balls. (**a**) Original structure (1CX0) and original density. (**b**) Reverse GU wobble base pair in the rebuilt structure (4PR6). U20 now makes hydrogen bonds with G25, and the positive difference density peak has disappeared.

Biochemical studies (10) and the structure of the deoxy-inhibited ribozyme 3NKB indicated a reverse wobble base pair between G25 and U20. In the original 1CX0 structure (Figure 4(a)), the bases of this potential pair are too far apart for hydrogen bonding, and a positive difference density peak is seen near U20. The backbone of U20 forms two hydrogen bonds: one with the backbone of C22, and another weak one with C75, but C22 clashes with both U20 and C75. By using the single-residue version of ERRASER, we were able to create a new model for U20 that now forms the reverse-wobble hydrogen bonds, gets rid of the difference density peak (Figure 4(b)) and fixes the C22 clashes, resulting in a full set of hydrogen bonds matching those present in 3NKB. Improving the GU base pair geometry also helped uncover a water molecule that stabilizes tertiary interactions between the backbones of C21, G62 and C63.

Overall, the rebuilt cleaved structure does not have any geometry or ribose pucker outliers, and has fewer unrecognized backbone conformations. The clashscore is reduced to 0.75, placing the new structure in the 100th percentile for its resolution range. The final *R* = 19.85% and *R*_free_ = 24.08%, improved by 4.75 and 3.82%, respectively. Table 1 shows the complete MolProbity statistics for the original (PDB ID: 1CX0) and the rebuilt structure (PDB ID: 4PR6). Supplementary Figure S1 shows the improvement in outlier markup on the 3D structure, and Supplementary Table S1 lists all backbone conformers and their ‘suiteness’ quality.

**Table 1.**
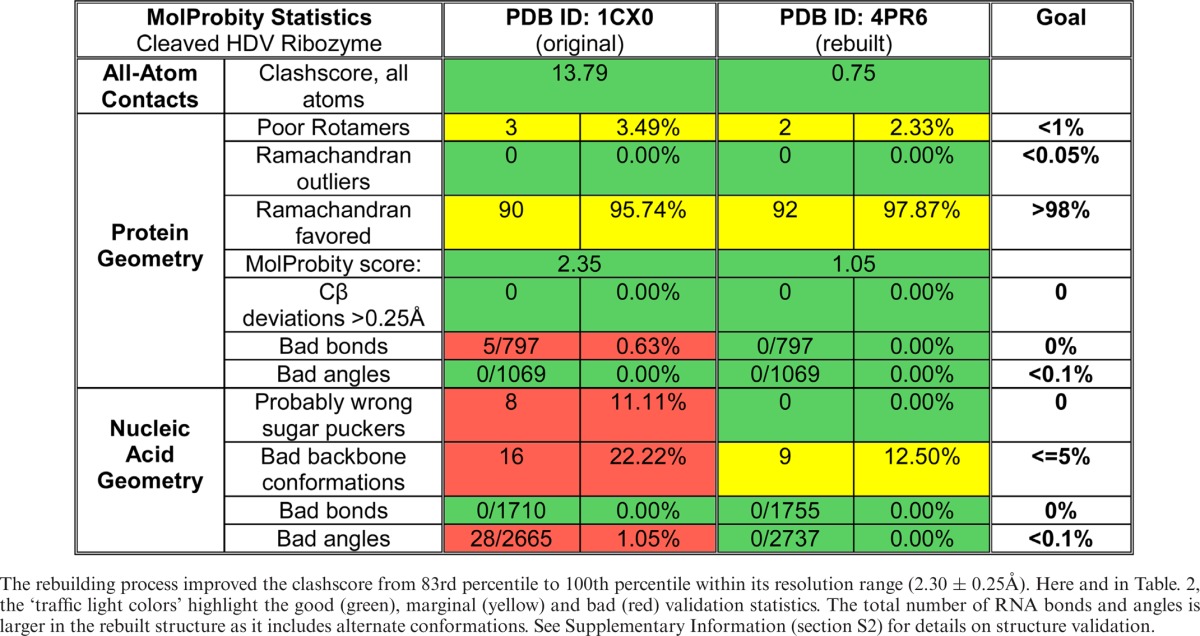
MolProbity statistics for the original cleaved structure (PDB ID: 1CX0) and its rebuilt version (PDB ID: 4PR6)

### Rebuild of the cis-acting C75U-inhibited 1VC7 structure

To prevent catalysis in the crystal structure of the HDV ribozyme, the catalytic residue C75 was mutated to a uracil. The structure of this catalytically inactive mutant was solved in the presence of a variety of metal ions that bind to the active site. The structure with a strontium ion in the active site (PDB ID: 1VC7) (16) contains, and shows, two additional residues upstream of the cleavage site (the ‘upstream nucleotides’) as compared to the cleaved structure: adenine at position -2 and uracil at position -1, with the cleavage site between residue -1 and 1 (Figure 2).

The C75U-inhibited structure 1VC7 was solved at 2.45Å resolution and has *R* = 23.91% and *R*_free_ = 26.40%, as calculated by PHENIX with anisotropic B factors from the original TLS refinement. It has a clashscore of 21.7, 61st percentile for its resolution range, and the bound U1A-RBD protein has a 95th percentile on MolProbity score. The RNA chain has 12/74 ribose pucker outliers, 23/74 unrecognized backbone conformations and six bond-length and 29 (1%) bond-angle outliers >4*σ*, three of which are >10*σ*.

After several cycles of automated rebuilding using tools described before, evaluation of their output, and sporadic manual rebuilding, alternated with rounds of refinement in PHENIX, the structure was significantly improved. All geometry and ribose pucker outliers were corrected, including 10 ribose puckers that were misfit as either C3′-endo or O4′-endo and now corrected to C2′-endo. The pucker corrections led to significant changes in the backbone conformers for a number of suites; 11 suites changed from **!!** to a recognized backbone conformer. The total number of unrecognized backbone conformations was reduced from 23 to 11 (see Supplementary Table S2). The following sections describe the specific changes most significant for the HDV ribozyme structure and function.

#### Alternate conformations for A16

**Figure 5.**
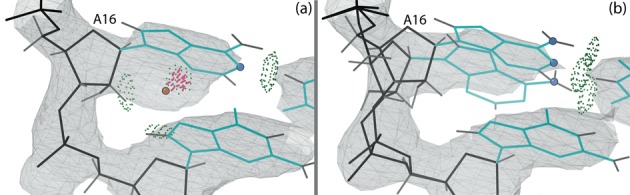
Base and backbone of residue A16 for the C75U-inhibited structure, with 2Fo-Fc electron density at 1*σ* contour level. The hydrogen-bonded heavy atoms of the base of A16 are highlighted as atom-colored balls. Here and in later figures, hydrogen bonds are shown as green dotted pillows and clashes as hotpink spikes. (**a**) A16 in the original structure (1VC7) and original density. The base of A16 clashes with a water molecule (peach ball). (**b**) The two alternate conformations for A16 in the rebuilt structure (4PRF) and new density.

Figure 5(a) shows the model and electron density for residue A16 in the original structure, which has a water molecule clashing with the base. Visual examination of the density suggested that a different conformation of the base would fit the density better, along with deletion of the water molecule. Indeed, the single-residue rebuild option in ERRASER modeled this conformation, although it results in a net loss of one hydrogen bond. The modeling of either conformation alone gave a positive difference density peak, but using both conformations together as alternates (Figure 5(b)) eliminates the difference density and matches the 2Fo-Fc density better for both base and backbone. Both alternates occupy the site equally, with the original conformation (alt A) refined to 0.51 occupancy and the new conformation (alt B) to 0.49 occupancy. A16 is involved in a Watson-Crick AU base pair and is located at the end of helix P2, which stacks co-axially on helix P3. This location at the helix end makes it plausible that this canonical AU base pair is somewhat destabilized and can open and switch to an alternate conformation.

#### Flipping of G25 to form a reverse GU wobble base pair

**Figure 6.**
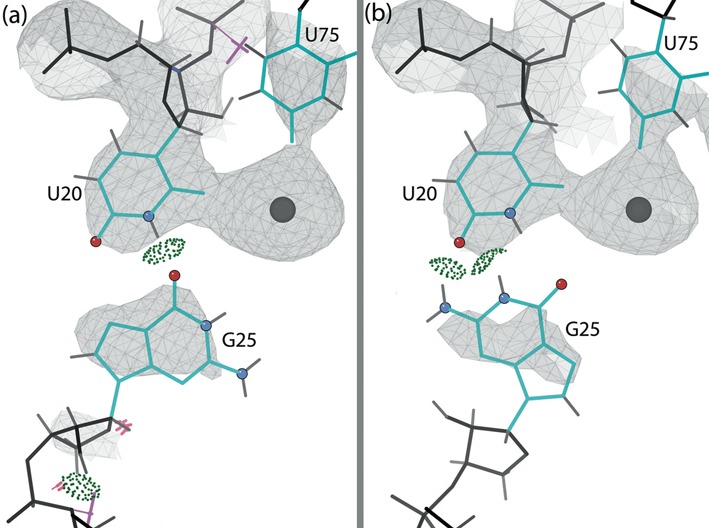
G25:U20 base pair and the metal ion (gray ball) in the C75U-inhibited structure in 2Fo-Fc electron density map at 1*σ* contour level. The hydrogen-bonded heavy atoms of G25 and U20 are highlighted as atom-colored balls. Here and in later figures, ribose pucker outliers are shown as magenta crosses. (**a**) Hoogsteen-WC base pair in the original structure (1VC7) and original density. Both G25 and U20 are ribose pucker outliers. (**b**) Reverse wobble base pair in the rebuilt structure (4PRF) and new density. Both ribose pucker outliers are corrected.

In the original structure, residue U20 interacts with the Hoogsteen edge of residue G25 forming only one hydrogen bond, and the RNA backbone model near G25 contains steric clashes and ribose pucker outliers (Figure 6(a)). ERRASER fixed the errors in the RNA backbone and, despite our exerting no direct control over the arrangement of the G25:U20 base pair, flipped the G25 residue, replacing the original Hoogsteen base pair by a reverse GU wobble base pair with two hydrogen bonds (Figure 6(b)). The electron density at this residue is very weak, especially for the backbone, and the base density is not clear enough to differentiate between the new and old conformations. Fitting both as alternates, or reducing the occupancy of the reverse wobble, worsened either the *R* values or the clashscore, and was therefore not accepted.

#### Active site corrections

**Figure 7.**
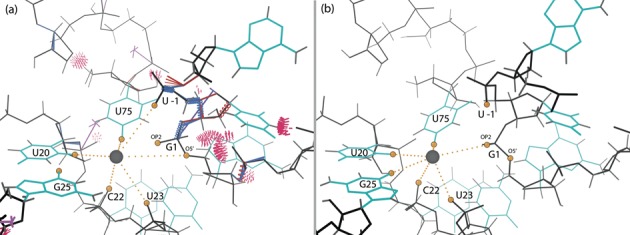
The active site of the C75U-inhibited ribozyme, with the catalytic metal ion (gray ball). The gold balls highlight the oxygen atoms in the metal ion neighborhood, and the dotted lines indicate metal ion interactions. Here and in later figures, bond-length outliers are shown as red and blue spirals, and bond-angle outliers are shown as red and blue fans. (**a**) Original structure (1VC7). The active site has a number of modeling errors. (**b**) The rebuilt structure (4PRF) active site, with all modeling errors corrected.

Inspecting the active site in the original 1VC7 structure revealed bad geometry, steric clashes and ribose pucker outliers (Figure 7(a)). The active site after rebuilding, with no clashes, geometry or pucker outliers, is shown in Figure 7(b). The important corrections made are discussed individually below.

The self-cleaving reaction of the HDV ribozyme depends on the catalytically active metal ion bound to the active site. Figure 7(a) and (b) show the metal ion (strontium here) and the nearby oxygen atoms in the original and rebuilt active site, respectively. The O2 of U20 and O4 of U75 are unambiguously close ligands and do not move significantly. The OP2 of C22 stays at about 5Å distance and points toward the metal. The OP1 of U23 shifts closer, to 3.6Å. The other nearby oxygen atoms are on residues that changed conformation in the rebuilding. O5′ of G1 and OP2 of U -1 move away from the metal ion by almost 1Å; at >6Å away, we do not consider them as interacting. But now OP2 of G1 (pro-Rp oxygen of the scissile phosphate) is unambiguously available for interaction with the metal ion at 4.2Å, along with O6 of the flipped G25 that now faces the metal ion at 4.1Å (atom names according to the Recommendations of the IUPAC-IUB Joint Commission on Biochemical Nomenclature available at http://www.chem.qmul.ac.uk/iupac/misc/pnuc2.html). Overall, the rebuilt structure has six oxygen atoms with distances <5Å from the metal ion. Active-site waters are not clearly visible, so it is difficult to evaluate the network of ligands. Supplementary Table S3 gives distances between the metal ion and the neighboring oxygen atoms in both the original and the rebuilt structure.

**Figure 8.**
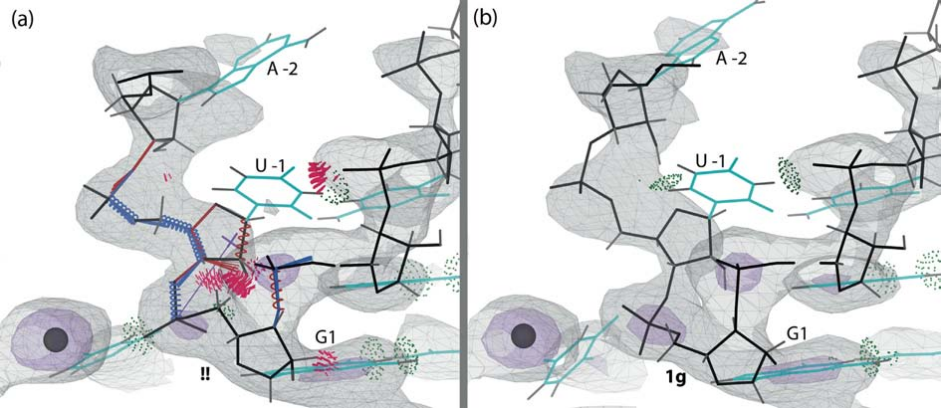
The position of the upstream nucleotides (A-2 and U-1) in the active site of the C75U-inhibited structure in 2Fo-Fc electron density map (contour levels 1*σ* as gray mesh, 3*σ* as purple mesh). (**a**) Original structure (1VC7) and original density. Modeling errors are highlighted. Suite 1 is an unrecognized backbone conformation (**!!**). (**b**) The rebuilt structure (4PRF) and new density. All modeling errors are corrected and suite 1 is now **1g**.

The upstream nucleotides in the original structure, and especially the critical backbone between U -1 and G1, show steric clashes, geometry outliers and a too-short (clashing) hydrogen bond between the base of U -1 and the backbone of C3 (Figure 8(a)). The improved model of the upstream nucleotides is shown in Figure 8(b). The N3 of U -1 and the non-bridging OP2 phosphate oxygen of C3 are now at an optimal hydrogen bonding distance. Suite U -1 is no longer an ϵ dihedral angle outlier and has an internal hydrogen bond, and suite G1 moves from **!!** to **1g**. An even better visual density fit for the upstream nucleotides resulted in a 0.3% higher *R*_free_ and was deemed unacceptable (see Supplementary Figure S3), thus leaving the backbone for A -2 and phosphate of U -1 somewhat less well fit to the density than the original structure. In compensation, however, the base for A -2 moves into electron density in the rebuilt model. The final model of the two upstream nucleotides, although not fit or refined with alternate conformations, shows electron density accounting for perhaps 80% of full occupancy, so the C75U mutation indeed succeeded in producing an uncleaved structure. The backbone suite between U -1 and G1 is now fit very satisfactorily, but the U -1 O2′ is still 9Å from the metal, and the position and orientation of the scissile bond is even less appropriate for catalysis than in 1VC7.

Because of the corrections made to the backbone of U75 and nearby residues, the position of the base U75 is slightly shifted in the rebuilt structure; it now more closely resembles the consensus position found in the related series of structures (16). Note that coordination of the metal ion by O4 of U75 would not be possible in the wild-type ribozyme, since the exocyclic amino group of a cytosine is present at this position as opposed to the keto oxygen of the uracil. This fact, as well as comparison with the deoxy-inhibited structure (3NKB) and single-atom modifications at residue 75 (20), suggests that the specific position of the mutated U75 in this uncleaved structure is probably not biologically relevant and the mutation may have prompted a change in the upstream nucleotide conformation as well (see Discussion).

Overall, the rebuilt structure has no geometry or ribose pucker outliers, and the number of unrecognized backbone conformations is reduced. The clashscore improved dramatically, from 21.8 to 0.76, placing the rebuilt structure at 100th percentile for its resolution range. The rebuilt structure was refined with two TLS groups, one for the RNA chain and one for the protein chain, and has *R* = 19.80% and *R*_free_ = 25.13%, an improvement of 4.11 and 1.27%, respectively. Table 2 shows the complete MolProbity statistics for the original (1VC7) and the rebuilt structure (4PRF). Supplementary Figure S2 shows the improvement in outlier markup on the 3D structures, and Supplementary Table S2 lists all backbone conformers and their ‘suiteness’ quality.

**Table 2.**
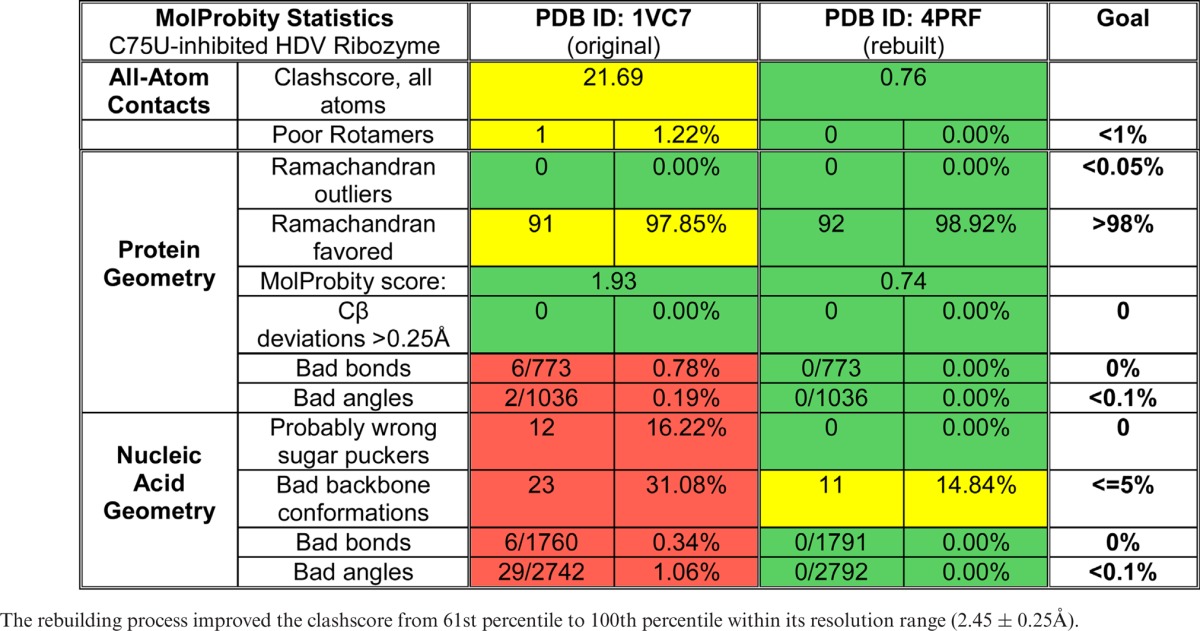
MolProbity statistics for the original C75U-inhibited structure (PDB ID: 1VC7) and its rebuilt version (PDB ID: 4PRF)

### Protein corrections

Crystallization of the cleaved and the C75U-inhibited structures was promoted by using the U1A-RBD protein as a crystallization module. It was shown that replacing part of the P4 stem-loop by the U1A binding sequence does not alter ribozyme cleavage activity in the presence or absence of U1A-RBD. Although the bound protein is irrelevant to catalysis, correction of model errors there helps improve the map elsewhere and the overall fit of the model to the experimental data, as reflected in both *R* and *R*_free_. Corrections to the U1A-RBD part of the structure were further validated by comparison with a higher resolution structure of the isolated U1A-RBD protein bound to its cognate RNA sequence (1.92Å, PDB ID: 1URN) (46).

#### Minor shifts to fix steric clashes

Our main interest is in making large corrections when the model is fit into the wrong local energy minimum. Fixing a clash or other outlier by small coordinate shifts is not important in itself and may not survive subsequent refinement. However, the process of trying such shifts is very important in order to determine that the outlier is not a symptom of a larger problem.

The Asn9 side chain in 1VC7 clashes with the adjacent backbone. Small changes in its side-chain dihedrals, plus a small backrub shift (45) were enough to alleviate the clash. The new conformation is further validated by 1URN and 1CX0/4PR6.

The Arg83 guanidinium in 1CX0 has a large steric clash with the well-fit O2 of PDB residue U150, persisting despite other improvements to the local structure. We used the side-chain rotator in KiNG to tweak the χ_2_ and χ_3_ angles to move Arg83 away from U150, and the new position held through refinement. Arg83 in 1URN does not bind the RNA, so its conformation cannot be compared.

#### Side-chain rotamer corrections

**Figure 9.**
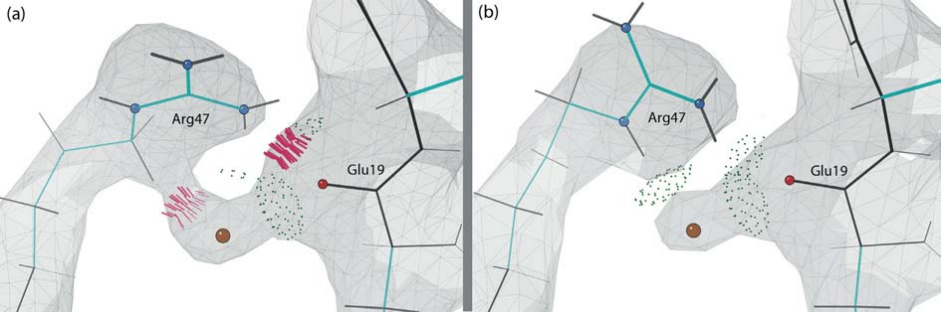
Arg47, Glu19 and nearby water molecule (peach ball) in the protein chain of the C75U-inhibited structure in the 2Fo-Fc electron density map at 1.2*σ* contour level. The hydrogen-bonded heavy atoms of Arg47 and Glu19 are highlighted as atom-colored balls. (**a**) **mmm180** rotamer of Arg47 in the original structure (1VC7) clashes with both the water and the backbone of Glu19. (**b**) **mtp180** rotamer of Arg47 in the rebuilt structure (4PRF) now makes hydrogen bonds with both the water and the backbone of Glu19.

In 1VC7, the Arg47 side chain clashes with the well-fit carboxyl group of Glu19 and a nearby water molecule (Figure 9(a)). We flipped the guanidinium group over, changing the rotamer from ‘**mmm180**′ to ‘**mtp180**′ (**m**, **p** and **t** mean **m**inus, **p**lus and **t**rans χ angles). The new rotamer now fits the density better, forms hydrogen bonds with both Glu19 and the water (Figure 9(b)) and was found also to match 1URN.

The Leu69 in both 1CX0 and 1VC7 clashes with local backbone, and is also a rotamer outlier in 1CX0. Refinement, alone, merely nudged clash and rotamer scores just past the cutoff values (better for clashes, worse for rotamers), with no significant net improvement. In contrast, changing to the ‘**tt**’ rotamer and then refining removes both clashes entirely, puts the χ angles in a highly favored range and matches the 1URN conformation.

In 1CX0, Ile33 clashes with the neighboring Phe34. Clear positive and negative density peaks suggested a ‘**pt**’ rotamer rather than the original ‘**mp**’ rotamer, which would then match 1VC7/4PRF. After the rebuild and refinement, the clash and the positive density are gone altogether and the negative density has mostly disappeared. This new conformation does not match the one found in 1URN, but that one itself has a clash, so it is unclear whether or not they really differ.

MSe72 in 1CX0 is originally fit as a ‘**mmt**’ rotamer, but the HE1 atom has a very large clash with the Arg36 side chain, which is also a rotamer outlier. The difference density showed a strong peak in the ‘**mmm**’ rotamer position, which would match both 1VC7/4PRF and 1URN. However, this position clashed with the previously modeled Ile33 position, which had to be refit first (as described above). We initially fit MSe72 with both alternate conformations, due to the extent of observed difference density in the area, but as the local model improved with further corrections, it became clear that modeling only the ‘**mmm**’ rotamer was necessary to fit the density. Modeling just this refit rotamer improved *R*_free_ by >0.25% and allowed Arg36 to move into a valid rotamer conformation.

## DISCUSSION

### Position of upstream nucleotides during the cleavage reaction

A defining characteristic of HDV-like ribozymes is the lack of sequence conservation for the upstream nucleotides (4,6). In addition, the C75U-inhibited structure showed an absence of any base-pairing interactions for the upstream nucleotides. As a result, the upstream nucleotides are somewhat disordered in the crystal structure, showing nearly full-occupancy 2Fo-Fc electron density for the backbone, but partial or no density for the bases. In fact, due to low electron density, the base of A -2 was not modeled in the other related structures (1SJ3, 1VBY, 1VBZ) except for 1SJF with cobalt hexamine bound (16). For these reasons, it has been difficult to accurately observe the position of the upstream nucleotides, despite the fact that their proximity to the catalytic center and the reactive nucleophile influences active site geometry.

Based on the position of the upstream nucleotides in the original C75U-inhibited structure, a catalytic mechanism was initially proposed that involved C75 acting as a general base, with a hinge motion around the bond O3′-P positioning the O2′, the phosphate group and the N3 atom of C75 for catalysis (16). Validation outliers in this region of the original structure have been corrected in the rebuilt structure 4PRF, with only moderate conformational changes to the upstream nucleotides (see the Results section for details).

**Figure 10.**
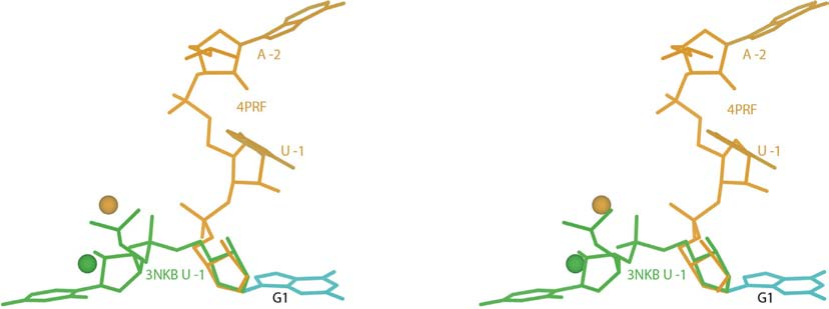
Stereo image of the upstream nucleotides (gold) in the rebuilt C75U-inhibited structure (4PRF) and the modeled upstream nucleotide U -1 and scissile phosphate (green) in the trans-acting deoxy-inhibited structure (3NKB) are shown, as superimposed on the base of residue G1. The positions of the corresponding metal ions in the active site are also shown. The two models for the upstream nucleotides are almost perpendicular to each other, and the two metal positions are nearly 4Å apart.

The 1.9Å structure of the deoxy-inhibited ribozyme 3NKB does not show significant electron density for the upstream nucleotide and it is, appropriately, not included in the coordinates. The authors used the conformation of the active site of the hammerhead ribozyme to model a plausible position for the upstream nucleotide U -1 and the scissile phosphate in the active site of the HDV ribozyme. Based on this model, they made structural interpretations to support the general-acid role of C75 in the cleavage reaction (21). In order to compare the model of the upstream nucleotides in the mutant versus the deoxy-inhibited structures, we recapitulated the model of U -1 and scissile phosphate in (21) by superimposing residue G1 of 3NKB and the equivalent residue in the hammerhead ribozyme (PDB ID: 2OEU (22)), followed by refinement to relieve a significant steric clash (see Supplementary Figure S4); the resulting conformation fits plausibly in its surroundings and was used for all further analyses. For the control test, modeling of the U -1 conformation from 4PRF into 3NKB and vice-versa resulted in large overlaps with the rest of the ribozyme in each case (see Supplementary Figures S6 and S7). Figure 10 compares the relative positions of the upstream nucleotides A -2 and U -1 in the rebuilt C75U-inhibited 4PRF and the U -1 and scissile phosphate modeled into the deoxy-inhibited 3NKB, as superimposed on residue G1. The positioning at the active site is very different in the two structures, with the two conformations for the upstream nucleotides coming in from nearly perpendicular directions.

**Figure 11.**
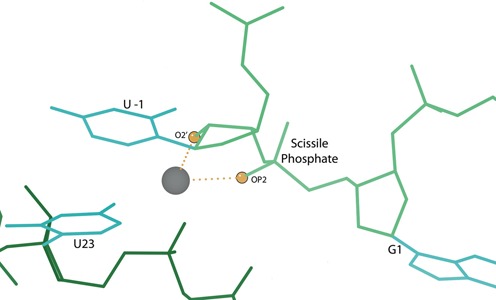
The position of G1, modeled U -1 and the scissile phosphate in 3NKB, based on the active site of the 2OEU hammerhead ribozyme structure (22). Modeled U -1 stacks with U23 and the dotted lines show the metal ion coordination by OP2 (pro-Rp) of the scissile phosphate and O2′ of U -1 (highlighted as balls).

The modeled position of U -1 and the scissile phosphate into 3NKB based on the hammerhead ribozyme makes three key interactions in the structure (Figure 11). The base of U -1 stacks on the base of U23 (which is near crystal contacts), and both O2′ of U -1 and OP2 (pro-Rp) of the scissile phosphate coordinate the Mg^2+^ in the active site. OP2 of the scissile phosphate had been shown to be important for the cleavage reaction (47), and this interaction of OP2 with the coordinated Mg^2+^ has been confirmed by phosphorothioate substitutions (25). This conformation also shows the potential of a hydrogen bond between the N3 atom of C75 and the O5′ of G1; this hydrogen bond is also observed in the rebuilt cleaved structure 4PR6. These interactions support the general-acid role of C75 (21) and make a good binding site for U -1, but as noted there is no clear density for it in the 2Fo-Fc map. This lack of density was attributed either to dynamic flexibility of the upstream nucleotides, or to the lowered occupancy due to a fraction of the substrate strand being cleaved in the crystal (10–50%, as reported in Supplementary Figure S2 of (21)) in spite of the deoxy substitution. Some positive difference density is seen in the region of the modeled U -1 conformation, but there is also an equal amount of negative difference density in that region (see Supplementary Figure S5), limiting the probable occupancy in that position to 10–20% or less. Although the modeled conformation seems reasonable, there is no direct crystallographic evidence for it. Several active-site differences from the cleaved 1CX0 structure have disappeared after the rebuilding, so that although 3NKB has the very major difference of an intact metal site, it is now somewhat more similar to the cleaved structure than previously apparent.

The rebuilt C75U-inhibited structure 4PRF has a C75U mutation of the essential base to prevent the cleavage reaction, and it has Sr^2+^ bound in the active site instead of Mg^2+^. The scissile phosphate interacts with the metal ion at OP2 of G1, but the two interactions made by the U -1 modeled into the deoxy-inhibited 3NKB (base-stacking on U23 and metal ion coordination by O2′) are absent. The hydrogen bond between N3 of C/U75 and O5′ of G1 (present in the cleaved 4PR6 and potentially in 3NKB) is also blocked by the scissile phosphate in 4PRF, and the 2′ hydroxyl of U -1 faces away from the metal ion. In addition, the coordination of Sr^2+^ by the O4 atom of U75 is an artefact of the C75U mutation, as an exocyclic amino group replaces the O4 atom in the wild-type ribozyme. However, the interaction of O6 of G25 and OP2 of the scissile phosphate with the metal ion is seen in the new model of the active site (along with moving away O5′ of G1), making the rebuilt 4PRF structure partially suggestive of the general-acid role of C75. These interactions are similar to the ones made by G25 and the modeled scissile phosphate in 3NKB. A molecular dynamics study that used the original C75U-inhibited 1VC7 structure as a starting model (restoring the C75 residue and the proper metal ion position) under different protonation states of C75 reported a spontaneous rotation of the upstream nucleotides to support the general-acid role of C75 (19). However, our attempts to model the rotated position into the rebuilt 4PRF active site failed. Overall, then, although the C75U mutation successfully inhibited cleavage, it is very likely that it distorted the active site, so that the upstream nucleotides in 4PRF do not represent the catalytic position.

The above analysis confirms the likelihood that the C75U mutation has distorted the active site in 4PRF, and highlights that the upstream nucleotide is not convincingly intact in 3NKB. Therefore it seems that neither strategy to inhibit the cleavage reaction and capture a molecular representation of the HDV ribozyme substrate has succeeded in achieving a good mimic of the pre-cleavage active site. Overall, we feel that the hammerhead analogy is almost certainly correct, but reiterate that there is not yet direct evidence from crystal structures.

### Role of the reverse G25:U20 wobble base pair

G25 and U20 are located in the active site and interact with groups essential for catalysis (C/U75 and the metal ion). In the original cleaved structure (1CX0), these bases were aligned as for a reverse GU wobble base pair, but too far apart for hydrogen bonding. In the original C75U-inhibited structure (1VC7), the Hoogsteen edge of G25 interacts with the U20 base. The rebuild for each of those structures (in independent ERRASER runs) resulted in a reverse G25:U20 wobble base pair: for 1CX0, small movements of U20 enabled the base-pair hydrogen bonds, whereas in 1VC7, G25 was flipped from *anti* to *syn* conformation (along with geometry and ribose pucker corrections). The reverse GU wobble base pair positions the O6 atom on G25 to interact with the active site metal ion in the C75U-inhibited structure, but no metal ion is bound in the cleaved structure. This suggests, in contrast to what was proposed earlier (21), that even though the reverse GU wobble base pair provides a good binding site for the metal ion, the metal ion is not required for formation of the base pair.

The 1.9Å 3NKB structure of the deoxy-inhibited ribozyme shows a reverse GU wobble base pair at this position with unambiguous electron density (21). The GU base pair lines one side of the active site and is implicated in binding the divalent catalytic metal ion. Several studies have found that mutating one or both these residues leads to significant decrease in the catalytic activity of the ribozyme, suggesting that these residues play a functional role in the catalytic mechanism (11,24,48). Also, residues G25 and U20 are conserved in catalytically active HDV-like ribozymes of various species, including the human CPEB3 gene (4,6,7). Molecular dynamic simulations starting from the original cleaved 1CX0 and the deoxy-inhibited structure 3NKB show that this reverse GU wobble base pair is stable both in the presence and absence of the metal ion (9). However, this base pair was not observed in the simulations starting from the structure 1SJ3 (one of the related structures to 1VC7 (16)), resulting in the conclusion that the reverse wobble base pair is not compatible with the C75U-inhibited structures. This is incorrect as the rebuilt structure 4PRF does have this reverse wobble base pair, and it is possible that the simulations were not able to flip G25 (along with the accompanying backbone changes) to make the required hydrogen bonds.

Biochemical and nuclear magnetic resonance studies show the presence of a reverse GU wobble base pair at this position in the cleaved structure, but also suggest that its formation is a post-cleavage event (48,49). This confirms the new reverse GU wobble base pair in the rebuilt cleaved structure, but is not consistent with the presence of this base pair in the rebuilt C75U-inhibited structure. The 2Fo-Fc electron density, in both the original and rebuilt C75U-inhibited structures, is reasonable for the G25 base, but not for its backbone; in fact, both structures show small negative difference-density peaks for the backbone of G25 and/or neighboring residues. This, combined with the fact of minimal and fragmentary electron density for residues 25–27, suggests that this region in the C75U-inhibited form can adopt multiple conformations including a reverse GU wobble base pair (modeled in the rebuilt 4PRF structure), which is the alternative stabilized after cleavage. This degree of conformational flexibility should not be considered unexpected or disturbing. However, while new methods have enabled our current work to improve the model in this important loop region, techniques are not yet up to determining a reliable conformational ensemble through fragmentary electron density.

### Interpretations of alternate conformations for U23 in the cleaved structure

The conformation of residue U23 in the original cleaved structure 1CX0 is very different from the C75U-inhibited 1VC7 structure. This had led to the conclusion that a significant conformational change occurs in residue U23 after the cleavage reaction (16), due to the absence of a metal ion in the active site to coordinate its phosphate. A close look at the electron density near U23 led us to model a second alternate conformation for this residue (see Results) similar to that seen in the inhibited structures. This suggests that release of the metal ion from the active site indeed makes U23 more flexible, but the residue can still sample a conformation similar to structures when cleavage is inhibited.

## CONCLUSION

New crystallographic, conformational-search and structure-validation tools now enable correction of existing RNA structures and better modeling of new ones. These tools identify significant local changes of RNA conformation that often move atoms by many Å and nearly always result in better scores for all categories of quality measures (*R*, *R*_free_, density fit, geometry, conformation, hydrogen bonding and all-atom sterics). For a small percentage of individual changes the criteria disagree, and acceptance should be based on conservative overall scientific judgment.

In this study, we have used these new techniques to correct and improve the cleaved and the cis-acting C75U-inhibited structures of the HDV ribozyme, which were originally solved over a decade ago. The changes include conformational correction of base, ribose, and backbone, fitting of multiple conformations, and identification of additional ions and waters. Our improvements, especially in the active site, alter biological interpretations about the catalytic mechanism of the HDV ribozyme made from the original structures. Our analyses and comparisons of the rebuilt structures with the trans-acting structure of the ribozyme (cleavage inhibited by removing the scissile 2′ hydroxyl) reveal that neither of the inhibited structures are a reliable representation of the pre-cleavage state or provide direct structural evidence for the currently accepted (26) hammerhead-like mechanism and general-acid role for C75. Therefore, further search for an inhibition strategy both thorough and non-perturbing would be worthwhile. This study also highlights the role these new tools can play in assisting crystallographers to make well-supported biological conclusions by helping build reliably accurate models.

## SUPPLEMENTARY DATA

Supplementary Data are available at NAR Online.

SUPPLEMENTARY DATA

## ACCESSION NUMBERS

4PR6, 4PRF.
