# Supplementary material for: New tools provide a second look at HDV ribozyme structure, dynamics and cleavage
Source: Nucleic Acids Res. 2014 Oct 17;42(20):12833–46. doi: 10.1093/nar/gku992 (PMC4227795; doi:10.1093/nar/gku992)
Supplement: SUPPLEMENTARY DATA [file supp_42_20_12833__index.html]

New tools provide a second look at HDV ribozyme structure, dynamics and cleavage — New tools provide a second look at HDV ribozyme structure, dynamics and cleavage — SUPPLEMENTARY DATA 

# New tools provide a second look at HDV ribozyme structure, dynamics and cleavage

## SUPPLEMENTARY DATA

**Files in this Data Supplement:**

- SUPPLEMENTARY DATA
- SUPPLEMENTARY DATA
