# Supplementary material for: New tools provide a second look at HDV ribozyme structure, dynamics and cleavage
Source: Nucleic Acids Res. 2014 Oct 17;42(20):12833–46. doi: 10.1093/nar/gku992 (PMC4227795; doi:10.1093/nar/gku992)
Supplement: SUPPLEMENTARY DATA [file supp_gku992_nar-01529-r-2014-File013.pdf]

SUPPLEMENTARY INFORMATION For

New Tools Provide a Second Look at HDV Ribozyme Structure, Dynamics, and Cleavage

Gary J. Kapral[1,4], Swati Jain[1,2,4], Jonas Noeske[3], Jennifer A. Doudna[3], David C. Richardson[1] and Jane S. Richardson[1,*].

[1] Department of Biochemistry, Duke University, Durham NC 27710, USA

[2] Program in Computational Biology and Bioinformatics, Duke University, Durham NC 27710, USA

[3.] Department of Molecular and Cell Biology, University of California Berkeley, Berkeley, CA 94720, USA

[4.] The authors wish it to be known that, in their opinion, the first two authors should be regarded as joint First Authors.

* To whom correspondence should be addressed. Tel: 1-919-684-6010; Fax: 1-919-680-6010; Email: jsr@kinemage.biochem.duke.edu

S1 Outlier markup for the cleaved and C75U-inhibited HDV Structures

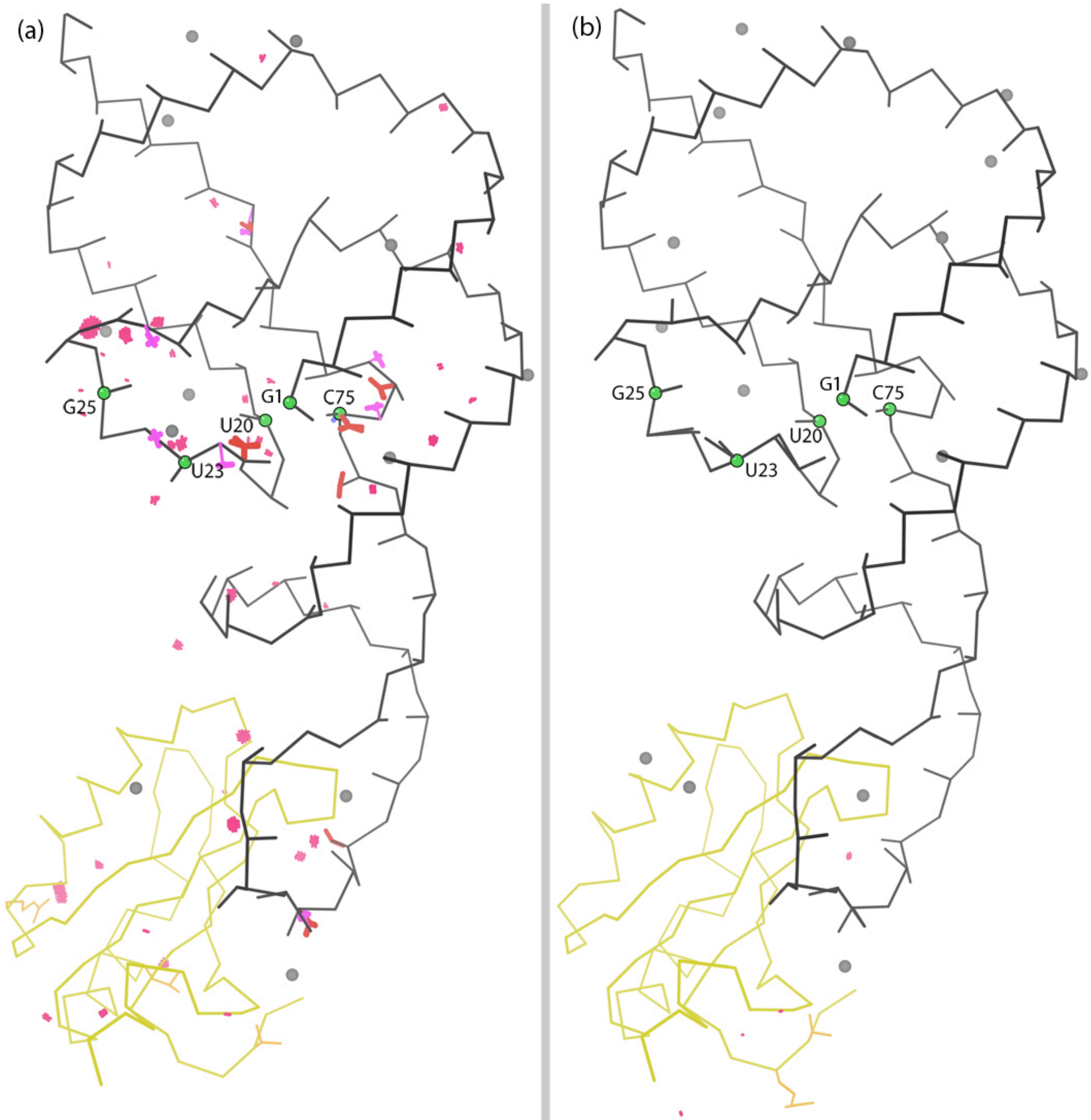

Figure S1: MolProbity (1) style outlier markup for the cleaved HDV Ribozyme structure. The RNA virtual backbone is in black and the protein main chain is in yellow. Here and in Figure S2, the outliers are marked as follows: steric clashes as pink spikes, ribose pucker outliers as magenta crosses, bond-length outliers as red and blue spirals, bond-angle outliers as red and blue fans, and rotamer outliers as gold side chains. (a) The original structure (PDB ID: 1CX0) (2) and (b) The rebuilt structure (PDB ID: 4PR6).

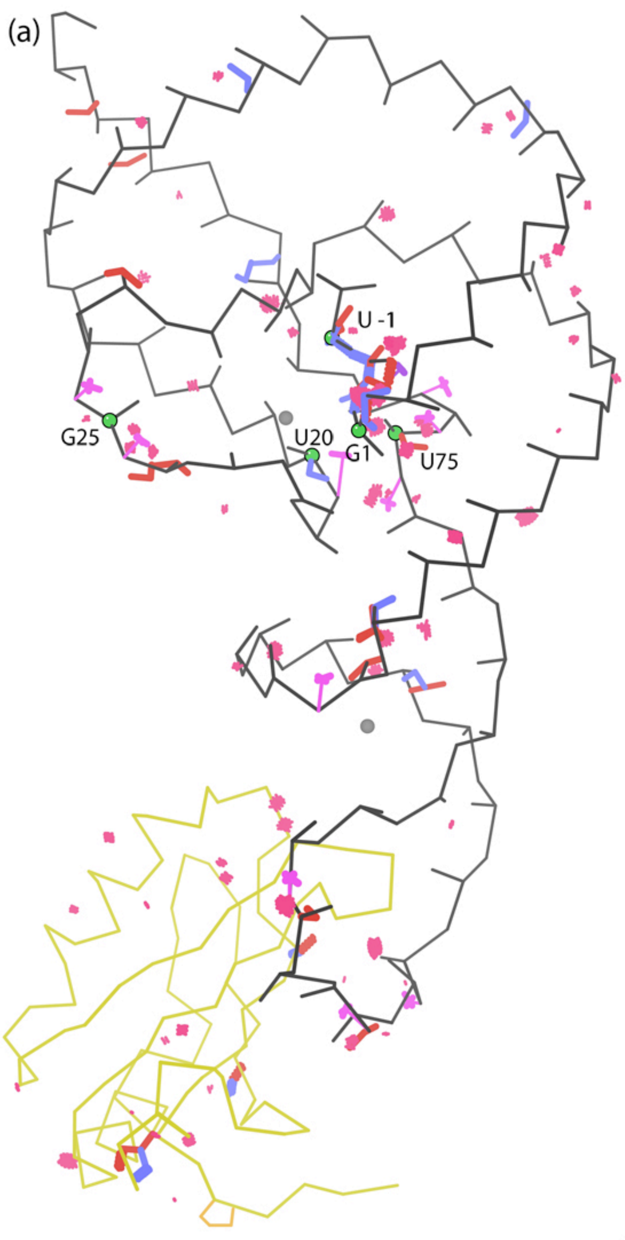

Figure S2: MolProbity (1) style outlier markup for the C75U-inhibited HDV Ribozyme structure. The RNA virtual backbone is in black and the protein main chain is in yellow. (a) The original structure (PDB ID: 1VC7) (3) and (b) The rebuilt structure (PDB ID: 4PRF).

S2 Details on Structure Validation

To assess model-to-data match we used R, R_{free}, and difference electron density. To access model quality, we used MolProbity (version 4.1) (1), a structure-validation web service that evaluates many features of RNA and protein models, or the equivalent functionality within PHENIX (4, 5). MolProbity uses Reduce (6) to add hydrogen atoms to the model, and Probe (7) to carry out all-atom contact analysis. Atomic overlaps of >0.4Å represent impossible steric clashes, and are reported both individually, and as '*clashscore*', calculated as the number of clashes per 1000 atoms. The model is assigned a percentile for the clashscore, relative to structures of similar resolution. MolProbity also analyses bond-lengths and bond-angles, and flags any residues that deviate by >4σ from the expected value as outliers. For protein models, the backbone is evaluated for Ramanchandran outliers and deviations in Cβ position. Protein side chains are evaluated for rotamer outliers. In addition Asn, Gln, and His side-chain ends are analysed, since they are often fit 180**°** backwards, and are flipped if necessary. The protein chain is given an overall MolProbity score (along with its percentile) taking into account the above-mentioned validation criteria.

Apart from geometry and all-atom contact analysis, MolProbity evaluates RNA chains for ribose pucker outliers and RNA backbone conformers. The ribose sugar in RNA almost always adopts one of the two pucker conformations: C3'-endo or C2'-endo. The ranges for the value of the δ dihedral angle (C5'-C4'-C3'-O3') are: 60°-105° and 125°-165° for C3'-endo and C2'-endo pucker respectively. To validate the modelled pucker for a particular residue, a perpendicular is dropped from the 3' phosphorous atom to the extended line of the glycosidic bond (C1'-N1/N9) vector. The length of the perpendicular is shown to be highly co-related with the pucker of the ribose ring: >2.9Å for C3'-endo and <2.9Å for C2'-endo pucker (8). If the length of the perpendicular and the value of the δ angle indicate different puckers, the residue is flagged as a ribose pucker outlier. In addition, the residues are also flagged if their ε dihedral angle (C4'-C3'-O3'-P_{+1}) value is outside the preferred range (155°-310°).

The sugar-phosphate backbone of the RNA chain is shown to be rotameric and most likely adopts one of the 54 recognized backbone conformers (9, 10). These backbone conformers represent favourable conformations of the sugar-to-sugar unit of the RNA backbone, called a *suite*. Each suite consists of seven dihedral angles: δ, ε, ζ of the previous residue and α, β, γ, δ of the current residue. Distinct clusters in seven dimensional space were identified as distinct backbone conformers, and were given a two-character name, each character representing the conformation of one of the hemi-nucleotides. As an example, **1a** represents the conformation of the RNA backbone in a standard A-form helix, and **1[** is the most common conformation adopted by an intercalation motif. For RNA backbone analysis in MolProbity and PHENIX, an automated program called Suitename analyses the backbone dihedral angles in the RNA chain and assigns a backbone conformer to each suite, or flags it as an outlier (denoted as **!!**) if it does not fall under any of the 54 recognized backbone conformers. The first step in this process is to place the suite in one of the 12 bins for $\delta_{-1}\delta\gamma$ angles (C3' or C2' for δ angles and **m**inus, **p**lus, or **t**rans for γ angle), and next to assign a particular backbone conformer to the suite within that bin. A value called *suiteness* is assigned to each suite (a number between 0 and 1, 0 for outlier suites), which provides a measure of how close it is to the centre of the backbone conformer cluster. The suites are also flagged as 'triaged' if any of the seven dihedral angles fall outside the preferred range of values (10).

S3 Backbone corrections in the cleaved and C75U-inhibited structures of the HDV ribozyme

The original cleaved structure of the HDV ribozyme (PDB ID: 1CX0) (2) has eight ribose pucker outliers (shown in red in Table. S1) and 16 unrecognized backbone conformations. The rebuilt structure (PDB ID: 4PR6) has no ribose pucker outliers, and the number of unrecognized backbone conformations was reduced from 16 to 9. Table. S1 shows the backbone conformers for the original and rebuilt cleaved structure. The pucker corrections were particularly impressive—hand refits and the use of RNABC were initially successful in correcting only one of these ribose puckers, residue 152, refitting the C3'-endo pucker as C2'-endo, while the other seven ribose puckers were each corrected to C2'-endo pucker only by using ERRASER. Correcting these ribose pucker outliers resulted in four suites changing to recognized backbone conformers: **1b** for 122, **1[** for 152, **2o** for 163, and **6p** for 164. The other three corrected ribose pucker outliers, 123, 127, and 167, remain outlier suites but have an improved fit to the electron density and no longer exhibit geometry outliers and steric clashes. This strategy also allowed six of the unrecognized backbone conformations to adopt known valid conformers. In total, 12 suites were changed from the original: seven of these are corrections from **!!** in the original to valid conformers, one is a change from **7d** to **!!**, and the other three change to different valid conformers. Some residues change more than once

during the process; for instance residue 122 goes from **1f** to **1t** to **1b**, depending on whether it has been through the full ERRASER protocol or just PHENIX refinement. The only residue to be changed into an apparent outlier is 110, which goes from **7d** to **!!**. Interestingly, the new conformation appears to be experimentally and sterically valid, with no geometry outliers, no clashes, and good fit to density. Thus, it represents a region of conformational space that is rarely sampled in existing structures, but which may be confirmed by future data to establish a new official backbone conformer.

Table S1: Backbone conformers for the cleaved HDV ribozyme, the original (PDB ID: 1CX0) and the rebuilt (PDB ID: 4PR6) structures. Residues are numbered according to the PDB file. Residues colored in red were ribose pucker outliers in the original structure, that were corrected in the rebuilt structure, leading to change in the backbone conformers for suite i and i+1.

	**PDB ID: 1CX0**			**PDB ID: 4PR6**		
**PDB Residue Number**	**$\delta_{-1}\delta\gamma$ bin**	**Backbone Conformer**	**suiteness**	**$\delta_{-1}\delta\gamma$ bin**	**Backbone Conformer**	**suiteness**
101	Incomplete	__	0.00	Incomplete	__	0.00
102	33 p	1a	0.881	33 p	1a	0.877
103	33 p	1a	0.930	33 p	1a	0.941
104	33 p	1a	0.877	33 p	1a	0.922
105	33 p	1a	0.935	33 p	1a	0.945
106	33 p	1a	0.884	33 p	1a	0.930
107	33 p	1a	0.898	33 p	1a	0.785
108	33 p	1a	0.845	33 p	1a	0.906
109	33 p	1a	0.860	33 p	1a	0.938
110	33 p	7d	0.426	33 t	!!	0.00
111	33 p	1a	0.700	33 p	1a	0.789
112	33 p	1a	0.905	33 p	1a	0.947
113	33 p	1a	0.929	33 p	1a	0.842
114	33 p	1a	0.661	33 p	1a	0.880
115	33 p	1a	0.806	33 p	1a	0.836
116	33 p	1a	0.947	33 p	1a	0.786
117	33 p	1a	0.889	33 p	1a	0.557
118	33 p	1a	0.921	33 p	1a	0.925
119	33 p	1a	0.847	33 p	1a	0.964
120	33 p	1a	0.197	32 p	1b	0.837
121	33 p	3d	0.122	23 p	8d	0.411
122	33 t	1f	0.568	32 p	1b	0.582
123	Triaged	!!	0.00	22 p	!!	0.00
124	Triaged	!!	0.00	Triaged	!!	0.00
125	Triaged	!!	0.00	23 t	6n	0.612
126	33 t	!!	0.00	33 t	!!	0.00
127	Triaged	!!	0.00	32 p	!!	0.00
128	Triaged	!!	0.00	23 m	0k	0.085
129	33 p	1a	0.636	33 p	1a	0.740
130	33 p	1a	0.691	33 p	1a	0.936
131	33 p	3d	0.559	33 p	3d	0.357
132	33 p	1a	0.915	33 p	1a	0.960
133	33 p	1a	0.830	33 p	1a	0.706
134	33 p	1a	0.916	33 p	1a	0.790
135	33 p	1a	0.843	33 p	1a	0.701
136	33 p	1a	0.923	33 p	1a	0.890
137	33 p	1a	0.943	33 p	1a	0.888
138	33 p	1a	0.620	33 p	1a	0.728
139	33 p	1a	0.851	33 p	1a	0.852

140	33 p	1a	0.978	33 p	1a	0.897
141	32 t	!!	0.00	32 t	!!	0.00
142	23 p	4g	0.054	23 p	4g	0.010
143	33 p	1a	0.814	33 p	1a	0.721
144	33 p	1a	0.895	33 p	1a	0.844
145	33 p	1a	0.918	33 p	1a	0.961
146	33 p	1a	0.88	33 p	1a	0.932
147	33 p	1a	0.914	33 p	1a	0.784
148	33 p	1a	0.948	33 p	1a	0.769
149	33 p	1a	0.917	33 p	1a	0.802
150	32 p	1[	0.771	32 p	1[	0.853
151	23 p	6g	0.422	23 p	6g	0.321
152	33 p	1m	0.209	32 p	1[	0.638
153	Triaged	!!	0.00	23 p	0a	0.626
154	33 p	1a	0.0815	33 p	1a	0.724
155	32 p	!!	0.00	32 p	!!	0.00
156	Triaged	!!	0.00	23 p	4g	0.574
157	33 p	1a	0.881	33 p	1a	0.922
158	33 t	!!	0.00	33 t	!!	0.00
159	33 p	1a	0.855	33p	1a	0.941
160	33 p	1a	0.956	33p	1a	0.886
161	33 p	1a	0.932	33p	1a	0.971
162	32 p	1b	0.698	33p	1b	0.692
163	23 m	!!	0.00	22 m	2o	0.281
164	Triaged	!!	0.00	22 p	6p	0.508
165	Triaged	!!	0.00	23 p	2a	0.760
166	33 p	1a	0.541	33 p	1a	0.146
167	Triaged	!!	0.00	32 t	!!	0.00
168	Triaged	!!	0.00	23 t	4n	0.303
169	33 p	1a	0.850	33 p	1a	0.981
170	33 p	1a	0.931	33 p	1a	0.743
171	33 p	1a	0.887	33 p	1a	0.949
172	33 p	1a	0.795	33 p	1a	0.742

The original C75U-inhibited structure of the HDV ribozyme (PDB ID: 1VC7) (3) has 12 ribose pucker outliers (shown in red in Table. S2) and 23 unrecognized backbone conformations. All geometry and ribose pucker outliers were corrected in the rebuilt structure (PDB ID: 4PRF), including 10 ribose puckers that were misfit as either C3'-endo or O4'-endo, and were corrected to C2'-endo. The number of unrecognized backbone conformations reduced from 23 to 11. Table. S2 shows the backbone conformers for the original and rebuilt structure. The pucker corrections led to significant changes in the backbone conformers for a number of suites. 11 suites changed from **!!** to a recognized backbone conformer: **1g** or 101, **1b** for 120, **8d** for 121, **1[** for 150, **6g** for 151, **0a** for 153, **5p** for 155, **4g** for 156, **2o** for 163, **6p** for 164, and **2a** for 165. Suite 152 changed from one intercalation conformer, **1m,** to another, **1[**, and suite 162 changed from **1a** to **1b** as the ribose pucker for both these residues was corrected from C3'-endo to C2'-endo. Suite 141 was incorrectly modelled as **1e**, but after its ribose pucker was corrected, it is now a **!!**. Residue 127 and 179 were both modelled as C3'-endo and were not flagged as ribose pucker outliers, but after rebuilding, both puckers changed to C2'-endo, as they fit the density better. This resulted in 2 suites moving from one backbone conformer to another: **7d** to **7p** for 127, and **1c** to **4n** for 168.

Table S2: Backbone conformers for the C75U-inhibited HDV ribozyme, the original (PDB ID: 1VC7) and the rebuilt (PDB ID: 4PRF) structures. Residues are numbered according to the PDB file. Residues colored in red were ribose pucker outliers in the original structure, that were corrected in the rebuilt structure, leading to change in the backbone conformers for suite i and i+1.

	PDB ID: 1VC7			PDB ID: 4PRF		
**PDB Residue Number**	**$\delta_{-1}\delta\gamma$ bin**	**Backbone Conformer**	**suiteness**	**$\delta_{-1}\delta\gamma$ bin**	**Backbone Conformer**	**suiteness**
99	Incomplete	__	0.00	Incomplete	__	0.00
100	Triaged	!!	0.00	23 t	!!	0.00
101	33 t	!!	0.00	33 p	1g	0.591
102	33 p	1a	0.010	33 p	1a	0.615
103	33 p	1a	0.745	33 p	1a	0.697
104	33 p	1a	0.689	33 p	1a	0.310
105	33 p	1a	0.611	33 p	1a	0.802
106	33 p	1a	0.412	33 p	1a	0.640
107	33 p	1a	0.629	33 p	1a	0.619
108	33 p	1a	0.754	33 p	1a	0.835
109	33 p	1a	0.825	33 p	1a	0.704
110	33 t	!!	0.00	33 t	!!	0.00
111	33 p	1a	0.661	33 p	1a	0.888
112	33 p	1a	0.691	33 p	1a	0.829
113	33 t	!!	0.00	33 t	1c	0.845
114	33 p	1a	0.863	33 p	1a	0.822
115	33 p	1a	0.963	33 p	1a	0.939
116	33 p	1a	0.795	33 p	1a	0.379
117	33 p	1a	0.760	33 p	1a	0.649
118	33 p	1a	0.851	33 p	1a	0.540
119	33 p	1a	0.935	33 p	1a	0.761
120	Triaged	!!	0.00	32 p	1b	0.586
121	Triaged	!!	0.00	23 p	8d	0.328
122	33 p	1c	0.520	33 p	1a	0.872
123	33 t	!!	0.00	32 m	!!	0.00
124	33 t	!!	0.00	22 t	!!	0.00
125	Triaged	!!	0.00	22 p	!!	0.00
126	Triaged	!!	0.00	23 t	!!	0.00
127	33 p	7d	0.033	32 p	7p	0.506
128	33 t	!!	0.00	23 t	6j	0.166
129	33 p	1a	0.839	33 p	1a	0.800
130	33 p	1a	0.874	33 p	1a	0.949
131	33 t	!!	0.00	33 t	!!	0.00
132	33 p	1a	0.750	33 p	1a	0.883
133	33 p	1a	0.885	33 p	1a	0.765
134	33 p	1a	0.634	33 p	1a	0.742
135	33 p	1a	0.809	33 p	1a	0.739
136	33 p	1a	0.807	33 p	1a	0.871
137	33 p	1a	0.919	33 p	1a	0.847
138	33 p	&a	0.956	33 p	1a	0.534
139	33 p	1a	0.804	33 p	1a	0.790
140	33 p	1a	0.934	33 p	1a	0.872
141	33 t	1e	0.010	32 t	!!	0.00
142	33 m	!!	0.00	23 p	!!	0.00
143	33 p	1a	0.180	33 p	1a	0.935

144	33 p	1a	0.802	33 p	1a	0.727
145	33 p	1a	0.900	33 p	1a	0.898
146	33 p	1a	0.745	33 p	1a	0.943
147	33 p	1a	0.868	33 p	1a	0.924
148	33 p	1a	0.899	33 p	1a	0.759
149	33 p	1a	0.964	33 p	1a	0.959
150	Triaged	!!	0.00	32 p	1[	0.908
151	Triaged	!!	0.00	23 p	6g	0.086
152	33 p	1m	0.566	32 p	1[	0.457
153	Triaged	!!	0.00	23 p	0a	0.750
154	33 p	1a	0.830	33 p	1a	0.875
155	33 p	!!	0.00	32 p	5p	0.010
156	Triaged	!!	0.00	23 p	4g	0.456
157	33 p	1a	0.565	33 p	1a	0.909
158	33 t	!!	0.00	33 t	!!	0.00
159	33 p	1a	0.921	33 p	1a	0.835
160	33 p	1a	0.913	33 p	1a	0.830
161	33 p	1a	0.789	33 p	1a	0.830
162	33 p	1a	0.494	32 p	1b	0.535
163	Triaged	!!	0.00	22 m	2o	0.423
164	Triaged	!!	0.00	22 p	6p	0.414
165	Triaged	!!	0.00	23 p	2a	0.576
166	33 p	1a	0.395	33 p	1a	0.546
167	33 t	!!	0.00	32 t	!!	0.00
168	33 t	1c	0.013	23 t	4n	0.177
169	33 p	1a	0.419	33 p	1a	0.883
170	33 p	1a	0.310	33 p	1a	0.120
171	33 p	1a	0.609	33 p	1a	0.834
172	33 p	1a	0.587	33 p	1a	0.907

S4 Metal ion coordination distances in 1VC7 and 4PRF

Table S3: The distances of the metal ion (Sr^{2+}) from the neighboring oxygen atoms are given (in Å) in the original (PDB ID: 1VC7) and the rebuilt (PDB ID: 4PRF) C75U-inhibited structures of the HDV ribozyme.

Atom Name	PDB ID: 1VC7 (Original)	PDB ID: 4PRF (Rebuilt)
O4 of U75	2.438	2.388
O2 of U20	3.13	3.086
OP1 of U23	4.16	3.624
OP2 of C22	5.076	4.981
O6 of G25	4.886 (facing away)	4.128
OP2 (pro-Rp) of G1	3.891	4.22
OP2 of U -1	5.221	6.227
O5’ of G1	5.328	6.267 (facing away)
