# Supplementary material for: New tools provide a second look at HDV ribozyme structure, dynamics and cleavage
Source: Nucleic Acids Res. 2014 Oct 17;42(20):12833–46. doi: 10.1093/nar/gku992 (PMC4227795; doi:10.1093/nar/gku992)
Supplement: SUPPLEMENTARY DATA [file supp_gku992_nar-01529-r-2014-File014.pdf]

SUPPLEMENTARY INFORMATION For

New Tools Provide a Second Look at HDV Ribozyme Structure, Dynamics, and Cleavage

Gary J. Kapral[1,4], Swati Jain[1,2,4], Jonas Noeske[3], Jennifer A. Doudna[3], David C. Richardson[1] and Jane S. Richardson[1,*].
[1] Department of Biochemistry, Duke University, Durham NC 27710, USA
[2] Program in Computational Biology and Bioinformatics, Duke University, Durham NC 27710, USA
[3.] Department of Molecular and Cell Biology, University of California Berkeley, Berkeley, CA 94720, USA
[4.] The authors wish it to be known that, in their opinion, the first two authors should be regarded as joint First Authors.
* To whom correspondence should be addressed. Tel: 1-919-684-6010; Fax: 1-919-680-6010; Email: jsr@kinemage.biochem.duke.edu

S5 A better fitting model of upstream nucleotides for the C75U-inhibited structure of the HDV ribozyme

Figure S3: An alternate model of the upstream nucleotides in the C75U-inhibited structure of the HDV ribozyme that visually fits the density better. The main difference is in the position of the base and the phosphate of residue A -2. The R_{free} for this refinement is 25.45%, which is almost 0.3% higher than PDB ID: 4PRF and hence was not acceptable.

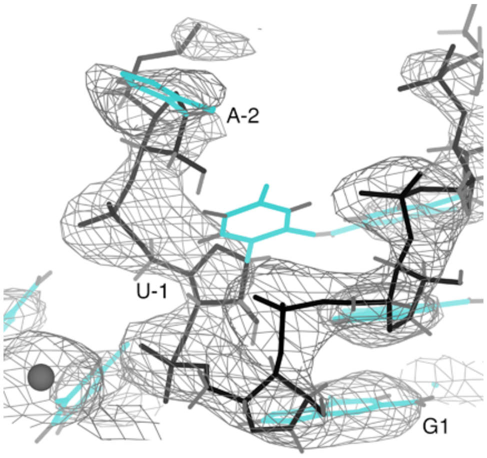

S6 Modelling of residue U -1 and the scissile phosphate into 3NKB

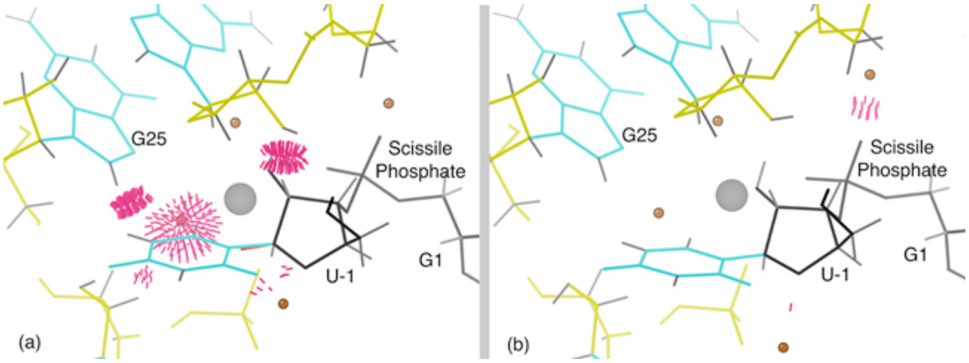

Figure S4: The active site of the hammerhead ribozyme (PDB ID: 2OEU) (11) was superimposed on the deoxy-inhibited structure of the HDV ribozyme (PDB ID: 3NKB) (12) to model the position of the upstream nucleotide U -1 and the scissile phosphate. To recapitulate the model, residue G1 from 3NKB was superimposed on residue 7 from 2OEU, along with deletion of three water

molecules. (a) Modelled U -1 clashes with the base of G25, backbone of U24 and G28, and a water molecule (peach ball) mediating the interaction between the metal ion (grey ball) and N7 atom of G25. The model was refined in PHENIX 1.8.2 (4) and the result is shown in (b) after 5 macrocycles of refinement. The refined model was used for all further analysis in this paper.

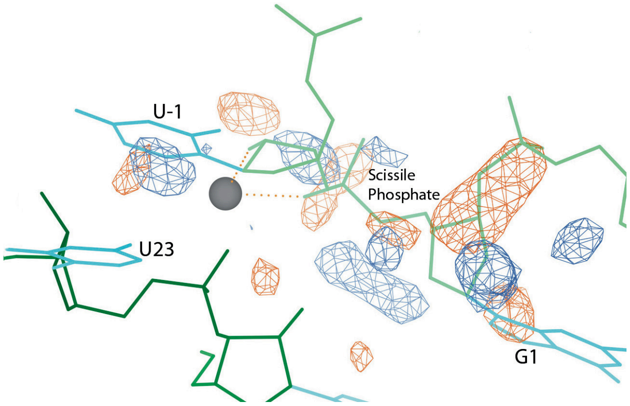

Figure S5: The position of G1, modelled U -1 and the scissile phosphate into PDB ID: 3NKB (12) based on the active site of the hammerhead ribozyme (PDB ID: 2OEU (11)) is shown with Fo-Fc difference density. Blue mesh shows the positive difference density peaks, and the orange mesh shows the negative difference density peaks, both at 3σ contour level. Modelled U -1 stacks with U23 and the dotted lines show the metal ion coordination by O2' of U -1 and OP2 (pro-Rp) of the scissile phosphate.

S7 Modelling of U -1 from 3NKB into 4PRF and vice-versa

Figure S6: The potential position of the modelled upstream nucleotide U -1 and scissile phosphate from PDB ID: 3NKB (12) (green) in the active site of PDB ID 4PRF (gold), as superimposed on the base of residue G1. The modelled U -1 overlaps with residue U23 in 4PRF.

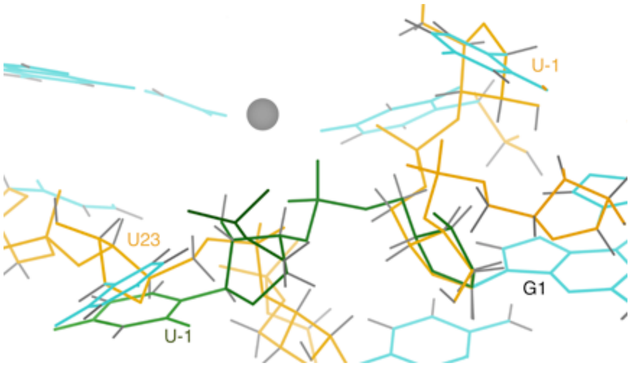

Figure S7: The potential position of the upstream nucleotides (A -2 and U -1) from PDB ID 4PRF (gold) in the active site of PDB ID 3NKB (12) (green), as superimposed on the base of residue G1. The upstream nucleotides overlap with residues C30, C75, A77 and A78 in 3NKB.

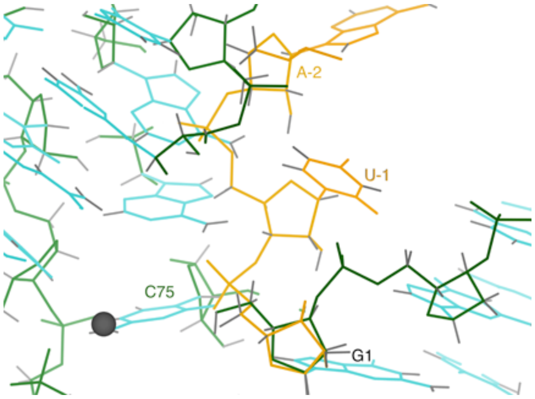

References

1. Chen, V.B., Arendall, W.B., III, Headd, J.J., Keedy, D.A., Immormino, R.M., Kapral, G.J., Murray, L.W., Richardson, J.S. and Richardson, D.C. (2009) MolProbity: all-atom structure validation for macromolecular crystallography. *Acta Cryst D*, **66**, 12–21.

2. Ferré-D'Amaré, A.R., Zhou, K. and Doudna, J.A. (1998) Crystal structure of a hepatitis delta virus ribozyme. *Nature*, **395**, 567–574.

3. Ke, A., Zhou, K., Ding, F., Cate, J.H.D. and Doudna, J.A. (2004) A conformational switch controls hepatitis delta virus ribozyme catalysis. *Nature*, **429**, 201–205.

4. Adams, P.D., Afonine, P.V., Bunkóczi, G., Chen, V.B., Davis, I.W., Echols, N., Headd, J.J., Hung, L.-W., Kapral, G.J., Grosse Kunstleve, R.W., et al. (2010) PHENIX: a comprehensive Python-based system for macromolecular structure solution. *Acta Cryst D*, **66**, 213–221.

5. Adams, P.D., Afonine, P.V., Bunkóczi, G., Chen, V.B., Echols, N., Headd, J.J., Hung, L.-W., Jain, S., Kapral, G.J., Grosse Kunstleve, R.W., et al. (2011) The Phenix software for automated determination of macromolecular structures. *Methods*, **55**, 94–106.

6. Word, J.M., Lovell, S.C., Richardson, J.S. and Richardson, D.C. (1999) Asparagine and glutamine: using hydrogen atom contacts in the choice of side-chain amide orientation. *J. Mol. Biol.*, **285**, 1735–1747.

7. Word, J.M., Lovell, S.C., LaBean, T.H., Taylor, H.C., Zalis, M.E., Presley, B.K., Richardson, J.S. and Richardson, D.C. (1999) Visualizing and quantifying molecular goodness-of-fit: small-probe contact dots with explicit hydrogen atoms. *J. Mol. Biol.*, **285**, 1711–1733.

8. Jain, S., Kapral, G.J., Richardson, D.C. and Richardson, J.S. (2014) Getting the Pucker Right in RNA Structures. *Computl Crystallogr Newsletter*, **5**, 4–7.

9. Murray, L.J.W., Arendall, W.B., III, Richardson, D.C. and Richardson, J.S. (2003) RNA backbone is rotameric. *Proc. Natl. Acad. Sci. USA*, **100**, 13904–13909.

10. Richardson, J.S., Schneider, B., Murray, L.W., Kapral, G.J., Immormino, R.M., Headd, J.J., Richardson, D.C., Ham, D., Hershkovits, E., Dean Williams, L., et al. (2008) RNA backbone: Consensus all-angle conformers and modular string nomenclature (an RNA Ontology Consortium contribution). *RNA*, **14**, 465–481.

11. Martick, M., Lee, T.-S., York, D.M. and Scott, W.G. (2008) Solvent Structure and Hammerhead Ribozyme Catalysis. *Chemistry & Biology*, **15**, 332–342.

12. Chen, J.-H., Yajima, R., Chadalavada, D.M., Chase, E., Bevilacqua, P.C. and Golden, B.L. (2010) A 1.9 Å Crystal Structure of the HDV Ribozyme Precleavage Suggests both Lewis Acid and General Acid Mechanisms Contribute to Phosphodiester Cleavage. *Biochemistry*, **49**, 6508–6518.
